# Supplementary material for: The Construction and Exploration of a Comprehensive MicroRNA Centered Regulatory Network in Foxtail Millet (Setaria italica L.)
Source: Front Plant Sci. 2022 May 6;13:848474. doi: 10.3389/fpls.2022.848474 (PMC9121102; doi:10.3389/fpls.2022.848474)
Supplement: Supplementary file 6 [file Data_Sheet_6.zip › Sit-miRNovel8_Seita.4G230500.1_201_TPlot.pdf]

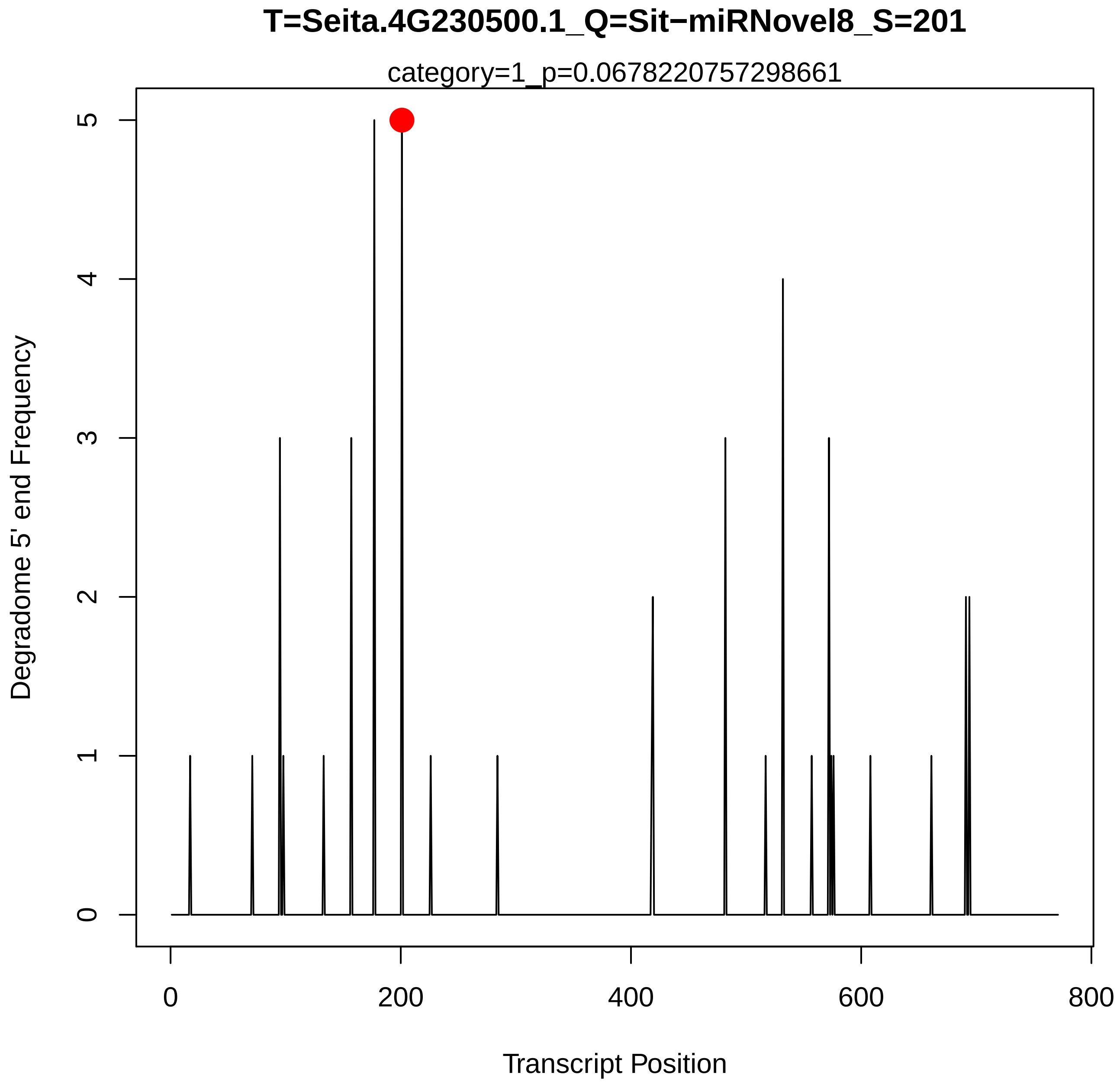

T=Seita.4G230500.1_Q=Sit−miRNovel8_S=201
category=1_p=0.0678220757298661
Degradome 5' end Frequency
Transcript Position
0
1
2
3
4
5
0
200
400
600
800
